# Olecranon Fracture in an Older Adult Treated With Locking Plate Osteosynthesis

**DOI:** 10.7759/cureus.18836

**Published:** 2021-10-17

**Authors:** Sreenivasulu Metikala, Nicholas G Poulos, Khalid Hasan, Madana Mohana R Vallem

**Affiliations:** 1 Orthopaedics, Virginia Commonwealth University School of Medicine, Richmond, USA

**Keywords:** elderly population, open reduction internal fixation, ground level fall, pre-contoured locking plate, geriatric patient, olecranon fractures

## Abstract

Although olecranon fractures are not uncommon in the geriatric population, there has been a considerable difference of opinion between surgical and nonsurgical treatments. Surgical treatment is usually deferred in the elderly, even for displaced olecranon fractures, because of inherent risks associated with poor bone quality and soft tissues, which often necessitate further surgeries. However, nonoperative treatment frequently results in an inability to regain full extension strength of the elbow, which can be disabling in select older adults with higher functional demands. We present an active older adult with a displaced olecranon fracture, who achieved a satisfactory result after open reduction and internal fixation (ORIF) using a low-profile locking plate.

## Introduction

Olecranon fractures in the elderly, unlike in young individuals, occur after a ground-level fall from standing height [1]. Typically, the fracture tends to displace (Mayo type II) due to the pull of triceps insertion resulting in a large fracture gap [2-4]. Despite fracture displacement, controversy exists between conservative and operative management among geriatric patients. Nonsurgical treatment in low-demand elderly patients has been shown to have acceptable outcomes while avoiding potential surgical complications [5-8]. However, healthy and active geriatric patients may have suboptimal outcomes with nonoperative treatment because of loss of elbow extension strength [9-10]. Therefore, recent attention has been directed towards surgical fixation in select elderly populations using newer generation osteosynthesis implants. We describe a case of displaced olecranon fracture in an older adult managed by open reduction and internal fixation (ORIF) by a low-profile locking plate, which led to satisfactory clinical and radiological outcomes.

## Case presentation

**Figure 1:**
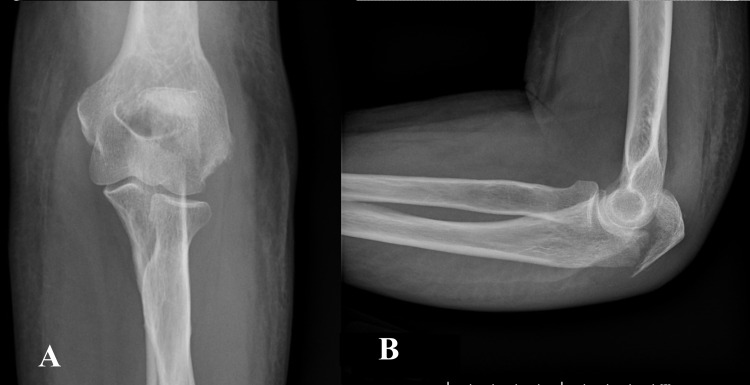
Plain radiographs of the injured left elbow. Anteroposterior (A) and lateral (B) radiographs of the injured elbow showing a displaced long oblique olecranon fracture along with cortical thinning and generalized increased bone radiolucency.

A 72-year-old right-hand dominant female tripped over a stick and fell on a sidewalk. She landed on the left side of the body resulting in immediate pain and swelling of the left elbow. She presented to the emergency department immediately and then to our clinic three days post-injury. Clinical examination revealed swelling accompanied by diffuse ecchymosis of the dorsal side of the left elbow and proximal forearm. The range of motion (ROM) at the elbow was limited due to pain. She could not actively extend the elbow against gravity. The results of the neurovascular examination were normal. Anteroposterior (Figure 1A) and lateral (Figure 1B) radiographs of the left elbow demonstrated a displaced long oblique fracture of the olecranon with a short proximal fragment and an additional intra-articular butterfly fragment. Also, thinning of cortices and generalized increased bone radiolucency with the altered trabecular pattern were evident in the radiographs.

The patient was a non-smoker and an active community ambulator. Her medical problems were hypothyroidism and type II diabetes mellitus, controlled on regular oral medications. Being medically well and active, she was recommended ORIF, which was performed as an outpatient procedure on day six post-injury.

**Figure 2:**
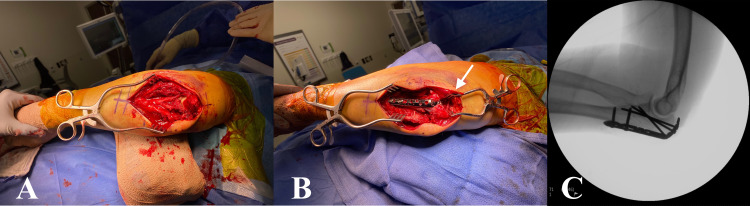
Intraoperative pictures. Exposure of olecranon fracture (A), application of proximal olecranon plate with triceps split (white arrow) at the insertion (B), and final fluoroscopic lateral view (C).

After successful general anesthesia, she was placed in a semi-lateral position using a beanbag on a standard operating table with her left upper extremity resting over the body. A single dose of 2 g of cefazolin was given intravenously as surgical prophylaxis. A pneumatic arm tourniquet was applied but not inflated. After sterile preparation and draping, a 10-cm midline posterior incision was made centering the fracture site with a smooth lateral curve around the tip of the elbow. Full-thickness flaps were created on either side. The ulnar nerve was identified and protected throughout the operation. The hematoma was evacuated, and fracture fragments were freshened (Figure 2A). Subtle comminution of articular and dorsal cortical margins was noted. The intraarticular butterfly fragment was found to be partially attached to the distal fragment. Direct reduction of the main fracture fragments was achieved by a pointed reduction clamp. Multiple 0.045-inch Kirschner wires (K-wires) were utilized for provisional fracture fixation. One of them was utilized as a joystick to anatomically reduce the butterfly fragment. The fracture reduction and articular congruity were verified by multiplanar fluoroscopic images. A pre-contoured low-profile proximal olecranon locking compression plate (DePuy Synthes, PA, USA) was placed along the dorsal surface. The triceps attachment was split in the midline to accommodate the head of the plate close to the bone (Figure 2B). Plate fixation was achieved with two 3.5-mm cortical screws into the ulnar shaft while three 2.7-mm (two cortical and one locking) screws were utilized proximally across the fracture line. Anatomic reduction, stability, and hardware position were verified by fluoroscopy (Figure 2C).

**Figure 3:**
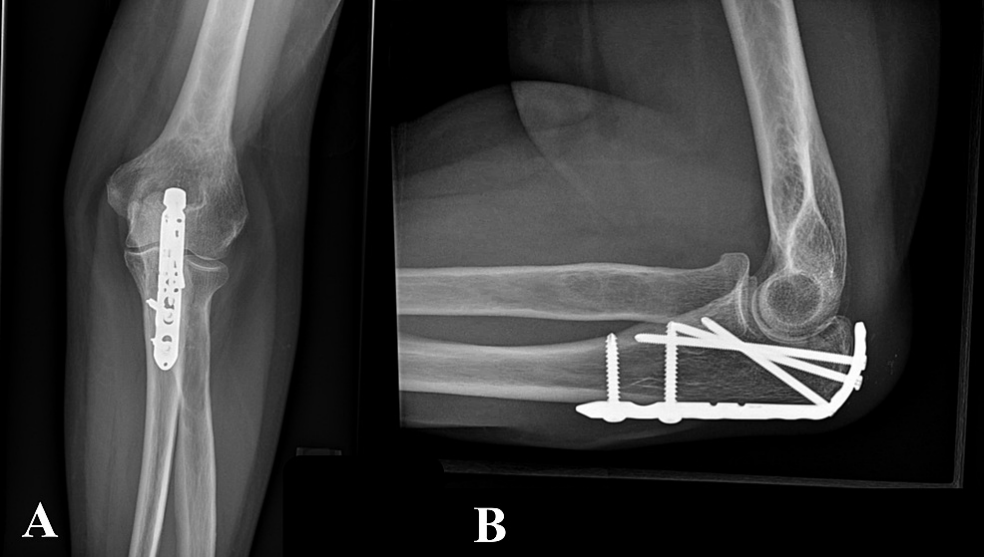
Follow-up radiographs of the left elbow at three months. Anteroposterior (A) and lateral radiographs (B) showing fracture healing in anatomic position and stable position of hardware.

The ROM was tested to check for any hardware impingement. The ulnar nerve was found to be stable in the cubital tunnel and therefore no transposition was attempted. The triceps split was meticulously repaired, followed by standard wound closure in a layered fashion. A well-padded long-arm posterior splint was applied over sterile dressings keeping the elbow in 70-degrees of flexion. The sutures were removed on the 10th postoperative day and the arm was placed in a sling. Outpatient physical therapy (PT) was initiated allowing active elbow flexion and gravity-assisted extension along with a weight-bearing restriction for up to six weeks postoperatively. The sling was then discontinued to allow active extension and begin isometric strengthening exercises followed by graduated resistance training as tolerated. At three months, she demonstrated 10-to-130-degrees of active elbow motion with no pain at the fracture site. Radiographs of the same visit showed healing of the fracture with a stable hardware position (Figures 3A, 3B).

She was, therefore, cleared to return all her previous activities with no restrictions. She was also discharged from PT with advice on regular home exercises.

**Figure 4:**
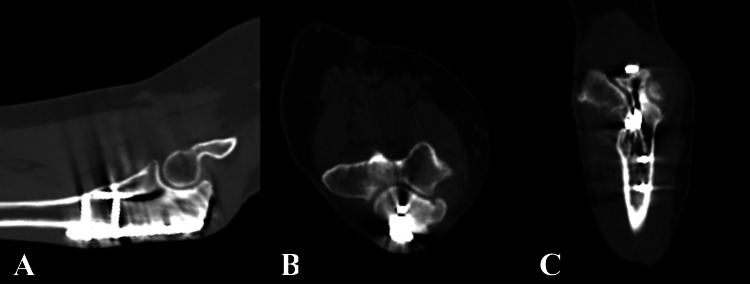
Computed tomography scan of the left elbow. Sagittal (A), axial (B), and coronal (C) slices confirm olecranon fracture union with no residual fracture lines.

She returned to the clinic two months later, approximately five months post-surgery, reporting intermittent pain and swelling of the proximal forearm overlying the distal portion of the plate. The follow-up radiographs did not show any hardware complications or stress fractures. After reviewing the treatment options, she had elected to pursue hardware removal. A computed tomography scan, performed before signing the patient up for this second surgery, had demonstrated no residual fracture lines confirming fracture union and stable position of hardware (Figures 4A-4C).

**Figure 5:**
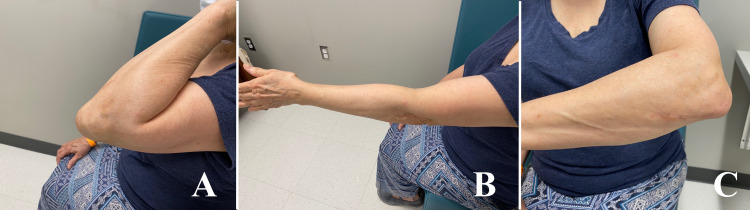
Follow-up clinical pictures at six months.

However, her surgery was delayed because of the onset of the third wave of coronavirus disease 2019 (COVID-19), causing all elective surgeries at our institution to be rescheduled. She subsequently reported gradual improvement in her symptoms of hardware irritation and finally elected against second surgery. At the recent six-month follow-up, she had regained full elbow ROM except for terminal 5-degrees of extension (Figures 5A-5C).

She had no pain in the elbow or proximal forearm, although the plate was still palpable over the ulnar shaft. Triceps strength was found to be equal and symmetric bilaterally. She returned to all her previous activities with no limitations and therefore cleared from our orthopedic trauma service with advice to return for hardware removal as needed.

## Discussion

The optimal treatment of displaced olecranon fractures in older adults is controversial. Nonoperative treatment comprises a couple of weeks of immobilization of the injured elbow in semi-extension followed by rehabilitation exercises. Low-demand elderly patients with multiple uncontrolled medical comorbidities and higher American Society of Anesthesiologists (ASA) scores may have acceptable outcomes with nonoperative management despite a high rate of nonunion and loss of elbow extension power [6,11-13]. Studies reported high complication rates in the elderly after ORIF of olecranon fractures because of poor bone quality, thin soft tissue envelops, and associated medical comorbidities [8,12]. However, elbow extension weakness affects several activities of daily living (ADLs), such as the ability to rise from a sitting position and mobilize with assistive devices. The loss of function and weakness maybe even more significant in active, independent, and healthy patients who routinely perform high-demand activities such as gardening, cooking, and daily exercises. The recent clinical studies on the management of displaced olecranon fractures in the elderly are summarized in Table 1.

**Table 1:** Summary of clinical studies on the management of displaced olecranon fractures in the elderly. LOE: level of evidence; F/U: follow-up; OES: Oxford Elbow Score; DASH: disability of arm, shoulder, and hand; ORIF: open reduction internal fixation; TBW: tension band wiring; MEPS: Mayo Elbow Performance Score.

Author (year)	LOE	No. of patients/mean age	Management	Mean F/U (months)	Outcomes
Duckworth et al. (2014) [12]	IV	43 (76 yr)	Nonoperative	72	Mean OES: 47; mean DASH: 2.9; mean extension loss: 18 degrees; nonunion 78%
Duckworth et al. (2017) [8]	I	19 (83 yr)	11 had ORIF (TBW/plate) and 8 had nonoperative	12	ORIF group with 81.8% complications versus a nonoperative group with 14.3% complications; no difference in mean DASH scores
Marot et al. (2018) [11]	IV	22 (88.8 yr)	Nonoperative	6	Mean MEPS: 95.26; mean DASH: 4.3; mean extension loss: 15 degrees; nonunion: 82%
Campbell et al. (2019) [14]	IV	36 (83 yr)	ORIF by plate	10	94% union; 11% reoperations
Wise et al. (2021) [10]	IV	36 (83.8 yr)	ORIF by plate	6	88.8% union at 8.9 weeks; 5.6% deep infections; 8.3% fixation failures; 13.9% minor complications

Anatomic reduction and stable fixation of a displaced olecranon fracture provide the best environment for early mobilization and rehabilitation, essential for geriatric patients. Given the increasing physical demands of our aging population, there has been a recent trend supporting ORIF of olecranon fractures in the select geriatric groups, who are medically well, active, and independent [5,10,14]. However, surgery is not without risk. The two most common surgical options include tension band wiring (TBW) and plate fixation. While TBW is generally applicable for a simple transverse fracture, the major drawbacks in the elderly with porotic bone include wire migration and secondary loss of reduction, skin impingement, and wound dehiscence [15]. A second operation, hardware removal, is quite common (up to 82%), leading to an additional financial burden [16]. The other complications, including nerve palsies and vascular injuries, have been reported due to the migration of the K-wires in the deeper planes [17,18]. Also, the studies that reported a higher postoperative complication rate in surgically managing geriatric olecranon fractures mostly utilized TBW as the surgical comparison [8,12].

Plate fixation is suitable for both stable and unstable fracture configurations. Although complications such as symptomatic hardware prominence and infection are possible even with plate fixation, it has lower secondary loss of reduction and hardware removal rates than TBW [5,10,14,19]. Locking plates are generally preferred to conventional nonlocking plates in elderly individuals, although there has been no clinical or biomechanical validation proving the superiority of one technique over the other [20]. The advantages of new generation locking plates, unlike conventional plates, include a pre-contoured design that can save time during surgery, a low profile with less chance of hardware irritation, and multiple screw options in the proximal portion of the plate. Nonetheless, locking plates do not completely obviate the need for a second surgery (hardware removal) and are more expensive than conventional plates. Our patient had initially suffered from symptoms of hardware irritation, which settled gradually avoiding a second surgery as of now.

## Conclusions

Operative fixation of an olecranon fracture by a low-profile locking plate is a reasonable option in an appropriately chosen geriatric patient. It permits early mobilization and restores active elbow extension power necessary for elderly patients with higher daily functional demands. However, hardware irritation is an inevitable limitation that may often necessitate a second surgery. The present case highlights the importance of shared decision-making based on the fracture displacement, patient’s age, comorbidities, and activity status.
